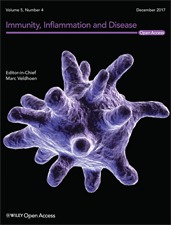# Issue Information

**DOI:** 10.1002/iid3.131

**Published:** 2017-11-17

## Abstract